# PI3-kinase has multiple functions in asexual blood stages of *Plasmodium falciparum*

**DOI:** 10.1038/s41598-025-01397-1

**Published:** 2025-05-14

**Authors:** Reem Al Monla, Maria Penzo, Alice Vallentin, Rakhee Lohia, Jeremy Vincent, Laurence Berry, Ana Rita Gomes, Rachel Cerdan, Kai Wengelnik

**Affiliations:** LPHI, CNRS, INSERM, University of Montpellier, Montpellier, France

**Keywords:** Artemisinin resistance, Haemoglobin uptake, Apicoplast, Lipid kinase, PtdIns3P, Vps34, Cellular microbiology, Parasitology

## Abstract

All symptoms of malaria are caused during the replication of the parasite *Plasmodium falciparum* in human red blood cells. The parasite digests the host cell cytoplasm in its food vacuole, a pathway of particular interest as drug target. The Vps34-type PI3-kinase in *P. falciparum* produces PI3-monophophate (PI3P) and has been linked to haemoglobin digestion, to resistance to the current first line antimalarial artemisinin and to biology of the apicoplast. Here we dissect the functions of *Pf*PI3-kinase by inducible deletion of its gene using the *loxP*-DiCre system and find that *Pf*PI3-kinase is essential for parasite survival. Mutant parasites accumulate undigested haemoglobin (Hb) confirming a defect in the pathway of Hb uptake and digestion, the most likely reason for parasite death. Some parasites are affected in apicoplast inheritance demonstrating that PI3P-dependent processes are important for apicoplast biology in *P. falciparum*. Finally, we discover that in PI3-kinase mutant parasites carrying a mutation conferring resistance to artemisinin, the lower amounts of PI3P correlate with lower levels of artemisinin resistance. We suggest that the reduced levels of PI3P in the PI3-kinase mutant dampen repair mechanisms like the autophagic processes normally associated with Kelch13 mutations, leading to a proteotoxic stress and to an increase in susceptibility to artemisinin.

**Supplementary Information:**

The online version contains supplementary material available at 10.1038/s41598-025-01397-1.

## Introduction

Malaria continues to weigh heavily on global health causing approximate 249 million cases and leading to over 608,000 deaths in 2022 according to the WHO World Malaria Report 2023^1^. The disease disproportionately affects the most marginalized populations in society and the African sub-Saharan region accounts for more than 90% of global cases and deaths. Malaria is caused by *Plasmodium* parasites with *P. falciparum* being the most virulent species^2^. The parasite is transmitted by blood feeding female Anopheles mosquitoes. After the initial non-symptomatic development in the liver, all disease symptoms are caused during the cyclic multiplication of the parasite in human red blood cells (RBCs). Parasites actively invades RBCs and form a parasitophorous vacuole, in which they grow and multiply by schizogony^3^. During this process, parasites progress through a series of morphological stages termed ring, trophozoites and schizonts, giving rise to up to 32 merozoite daughter cells at the end of a about 48-h cycle. Upon active egress the invasion of new RBCs initiates the next cycle. A small fraction of parasites differentiates into sexual stages that are transmissible to the mosquito vector during a blood meal allowing sexual reproduction and multiplication of the parasite and completing its life cycle^4^.

The host cells of *P. falciparum*, the mature human RBCs are particular cells because they do not harbour a nucleus or other organelles but carry large amounts of haemoglobin (Hb) which ensures the transport of oxygen in the body^5^. The parasite grows within a parasitophorous vacuole and consumes large portions of the RBC Hb through the uptake of the cytosol followed by Hb digestion in the parasite food vacuole^6^. The degradation produces free haem that is detoxified as hemozoin crystals, visible as dark pigment in the food vacuole. Many details of the mechanism of how host cell cytoplasm uptake occurs in *P. falciparum* are still speculative^7^. Host-cell invaginations that are observed by electron microscopy at the parasite periphery are the cytostome with a narrow neck surrounded by an electron-dense collar and the larger phagotroph^8^. How the Hb in these initial structures reaches the food vacuole where it is digested is still a matter of debate (reviewed in^7^.

Endocytic events and membrane trafficking are processes that often involve the presence of particular lipids such as phosphoinositides, phosphorylated derivatives of the structural membrane lipid phosphatidylinositol (PI)^9^. We previously established the phosphoinositide profile of the *P. falciparum* infected human RBC^10^ and discovered that the parasite produces surprisingly large amounts of PI3-monophosphate (PI3P), when compared to organisms, in which PI3P biology has been extensively studied like mammalian cells and yeast^9,11^. In these organisms the major roles of PI3P lie in two cellular processes: endolysosomal pathways and autophagy^11,12^, an intracellular degradation process that allows cells to recycle intracellular components to generate energy and provide building blocks^13^. PI3P is enriched in early endosomes and in autophagic vesicles and acts by recruiting proteins with domains specifically binding PI3P, the best studied being the PX- (Phox homology) and FYVE- (Fab1, YOTB, Vac1, EEA1) domains^14^. PI3P is produced by a class III PI3-kinase of the Vps34-type^12^.

In *P. falciparum* and the closely related apicomplexan parasite *Toxoplasma gondii* that often serves as model organism for apicomplexan cell biology, a single PI3-kinase of the Vps34-type produces predominantly PI3-monophosphate^10,15^. Counterintuitively to the high levels of PI3P, the genomes of *Plasmodium* spp. and *T. gondii* seem to encode very few proteins with domains known to bind PI3P and that could serve as actors for PI3P cell biology^16–18^. In *P. falciparum* expression of a fluorescently labelled PI3P-binding domain identified two distinct intracellular localisations for PI3P, around the food vacuole and at the apicoplast^10^. The apicoplast is present in many apicomplexan species and is an organelle with four membranes of algal origin derived from an ancestral secondary endosymbiosis event^19^. This organelle harbours few but essential metabolic processes^20^. In *P. falciparum* blood stages, only isoprenoid synthesis is essential and parasites can survive in the absence of their apicoplast if isopentenyl pyrophosphate (IPP) is supplied in the culture media^21^. Studies in *T. gondii* clearly established that PI3P is essential for the maintenance and inheritance of the apicoplast^15,16^. Conditional depletion of the TgPI3-kinase or masking the PI3P lipid by overexpression of a PI3P-sensor protein both led to parasite death through interference with apicoplast development and inheritance. In *P. falciparum* a functional link between PI3P and the apicoplast has so far not been shown. The main function of PI3P in *P. falciparum* was initially deduced from studies using two specific inhibitors of PI3-kinase, wortmannin and LY294002. Treatment interfered with food vacuole functions leading to the accumulation of undigested Hb within the parasite, and in electron microscopy images higher numbers of Hb-containing vesicles were observed^22^. A recent study using these inhibitors also revealed that the intracellular parasite degrades host cell alpha-spectrin through ubiquitination in a PI3-kinase-dependent way^23^.

Interest in the function of PI3-kinase in *P. falciparum* was boosted when Mbengue et al. discovered that cellular amounts of PI3P show a strong correlation with the level of resistance to the current first line antimalarial drug artemisinin^24^. Resistance is in many cases conferred by mutations in a protein termed Kelch13 (K13)^25–27^. It has been shown that both resistant clinical parasite lines as well as genetically modified K13 parasites show higher levels of PI3P^24^. One proposed mechanism for artemisinin resistance involves PI3P vesicle expansion and the unfolded protein response with K13 serving as substrate adaptor for E3 ligase thus leading to ubiquitination and proteolysis of PI3-kinase^24,28^. K13 mutations prevent PI3-kinase ubiquitination and degradation resulting in higher PI3P levels and resistant parasites. A different mechanism has recently been proposed suggesting that K13 interacts with several other proteins to form a K13-compartment. This compartment has recently been shown to correspond to the ring around the cytostomal neck^29,30^. The cytostome is involved in Hb uptake^31–33^. Upon endosomal-like vesicle transport to the food vacuole^32,34,35^, Hb digestion releases reactive haem that is thought to activate artemisinin for its antimalarial activity. Resistant parasites with mutated K13 have lower levels of K13 protein and Hb uptake is reduced leading to less release of haem that temporarily allows the parasite to persist during the transient peak of high levels of artemisinin^7,31,36,37^. In addition, PI3P has been suggested to play a role downstream of the K13 compartment in the endocytic process leading to the food vacuole^32^. Alternatively, PI3P-decorated vesicles carrying autophagy markers have been observed in K13 mutant parasites and were proposed to serve as a protective stress response^38^.

Here we generated a transgenic *P. falciparum* line that allows inducible gene deletion of the *Pf*PI3-kinase gene (PF3D7_0515300) using the *loxP*-DiCre system^39^. Efficient depletion of the PI3P lipid product was confirmed and PI3-kinase was found to be essential for parasite survival. The mutant was studied for the effect on apicoplast homeostasis, food vacuole functions and artemisinin resistance, all of which were indeed found to be linked to PI3-kinase activity.

## Results

### Generation of transgenic parasite lines with a floxed PI3-kinase locus

**Fig. 1:**
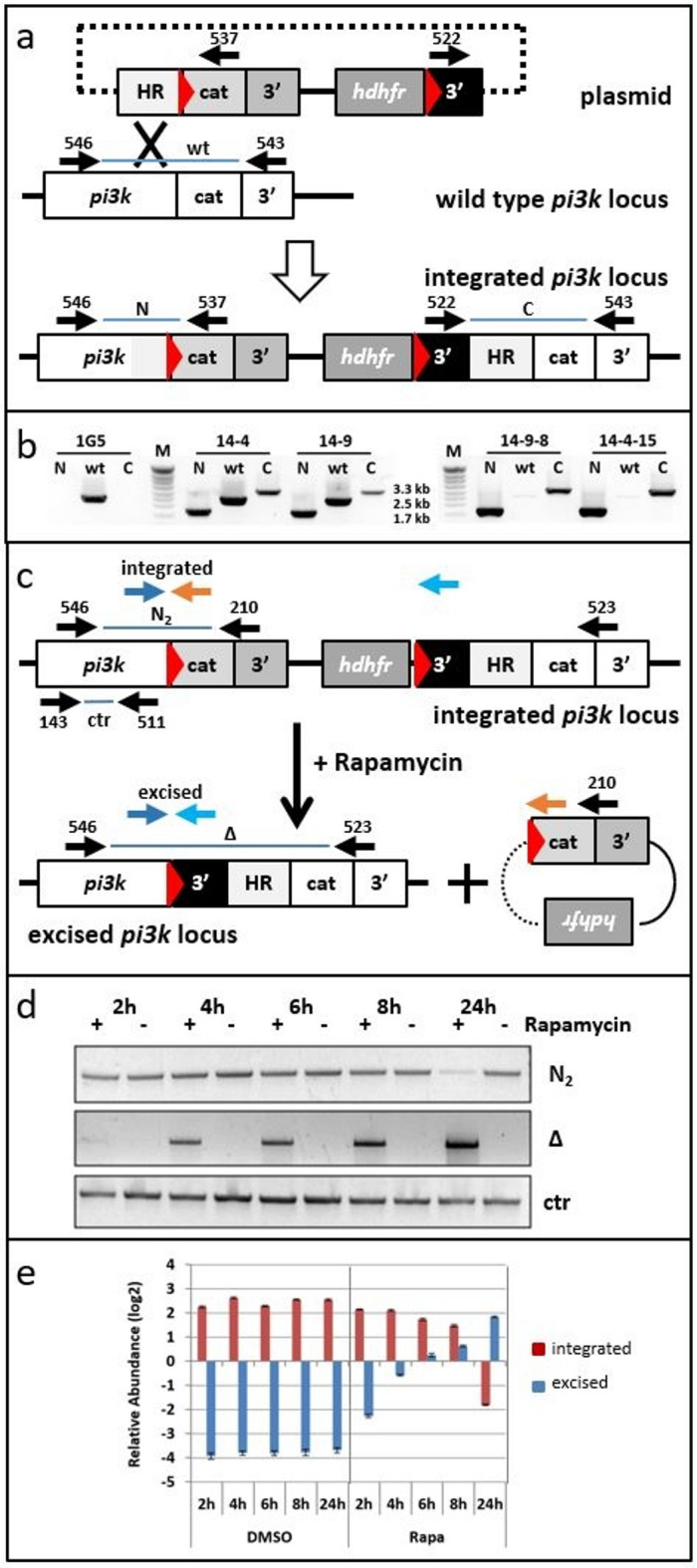
Generation of a parasite line with the catalytic domain of *pi3k* flanked by *loxP* sites. (**a**) Schematic representation of the transfection plasmid, the genomic *pi3k* locus and the resultant recombinant locus with the insertion of an in-frame *loxP* site upstream of the PI3K catalytic domain (cat). Red triangles represent *loxP* sites; HR: homology region; 3′: 3′UTRs of different genes: white: *Pfpi3k*, grey: *Pbdhfr/ts*, black: *Pfhsp86*. Primer positions with their identifiers and amplification products are indicated. The dotted line indicates that circular DNA was used for transfection but does not represent DNA sequences. Scheme not drawn to scale. (**b**) Genotyping of parasite populations and parasite clones. Single homologous recombination leading to integration could be detected for two independent transfections 14-4, and 14-9. Both populations were cloned and the genotype of clones 14-9-8 and 14-4-15 are shown. 1G5 is the parental DiCre-expressing strain. M: DNA size marker. Full-size gels are presented in Supplementary Fig. S5. (**c**) Schematic representation of the floxed *pi3k* locus before and after Cre-mediated recombination. Upon addition of rapamycin, DiCre is activated leading to the excision and circularisation of the sequences between the *loxP* sites excising the catalytic domain of *pi3k*. Black arrows indicate the positions of PCR primers used in panel (**d**), blue and red arrows correspond to qPCR primers used in panel (e). (**d**) Kinetics of recombination. DNA of clone PF14-9-8 was extracted at the indicated time points after rapamycin treatment (100 nM) at the ring stage and used for the PCR reactions indicated in panel (**c**). Excised locus could be detected early and only upon addition of rapamycin while reduction of complete (integrated) *pi3k* locus showed a delayed kinetics. Fragment sizes: N_2_: 2.5 kb, Δ: 2.3 kb, ctr: 1.9 kb. Full-size gels are presented in Supplementary Fig. S5. (**e**) Quantitative PCR analysis of the same DNA samples as in panel (**d**). The primers used are indicated in panel (**c**). Results were expressed as the ratio of the amplification of the target gene over the reference gene with log2 scale.

We had previously shown that the PI3-kinase gene in the rodent malaria parasite *P. berghei* could not be disrupted by double homologous recombination^10^. The *Pbpi3k* locus could be targeted but we did not obtain transgenic parasites when we attempted to tag the kinase at the C-terminus with either a triple hemagglutinin epitope (3HA) or green fluorescent protein (GFP) indicating that disrupting or even modifying the *Pbpi3k* gene at its extreme 3′ end was detrimental for *P. berghei* during its intraerythrocytic development^10^. In *P. falciparum*, targeted *pi3k* gene disruption by selection-linked integration has also not been achieved^17^. Here, in order to disrupt the *pi3k* gene in *P. falciparum* (PF3D7_0515300) we chose a conditional gene knock-out approach using the DiCre system in which a rapamycin inducible split version of the Cre recombinase mediates specific recombination between two *loxP* sites. We inserted one *loxP* site in frame in a poorly conserved sequence upstream of the region coding for the catalytic domain (Fig. S1). The second *loxP* site was placed in the plasmid backbone downstream of the hDHFR cassette (Fig. 1a) and the construct transfected in the DiCre-expressing 1G5 line^39^. Transgenic DiCre populations were subjected to multiple rounds of drug cycling to enrich in parasites that had integrated the transfection construct into the *pi3k* locus by single homologous recombination and cloned by limiting dilution. One clone was obtained from each of two independent transfections and named PF14-9-8 and PF14-4-15. PCR analysis detected recombination at the *pi3k* locus in these clones while wild type *pi3k* locus was absent (Fig. 1b) confirming that insertion of a *loxP* site within the *pi3k* coding sequence was tolerated.

### Rapamycin-inducible deletion of the catalytic domain of PI3-kinase gene

We then analysed Cre-mediated recombination upon the addition of rapamycin to sorbitol synchronized ring stage cultures of clone PF14-9-8 (Fig. 1c, d). PCR fragments that can only be amplified after *loxP*/Cre recombination were identified as early as 2 h after addition of rapamycin and increased over time. A complementary PCR amplification specific for the full length *pi3k* sequence that was expected to become less abundant with progressing Cre-mediated excision of the 3′ sequence of the PI3K gene, diminished with delayed kinetics in these semi-quantitative PCR reactions. Closer analysis by qPCR of the same genomic DNA samples confirmed that addition of rapamycin rapidly induced DiCre recombination and proceeded over 24 h reaching a 46-fold increase for the excised gene and a 20-fold decrease of the integrated construct (Fig. 1e). It also confirmed that DiCre recombination did not happen without rapamycin. In conclusion, recombination of the floxed catalytic domain of the *pi3k* gene was initiated promptly and exclusively following the addition of rapamycin.

### Survival of the inducible PI3-kinase mutant parasites

**Fig. 2:**
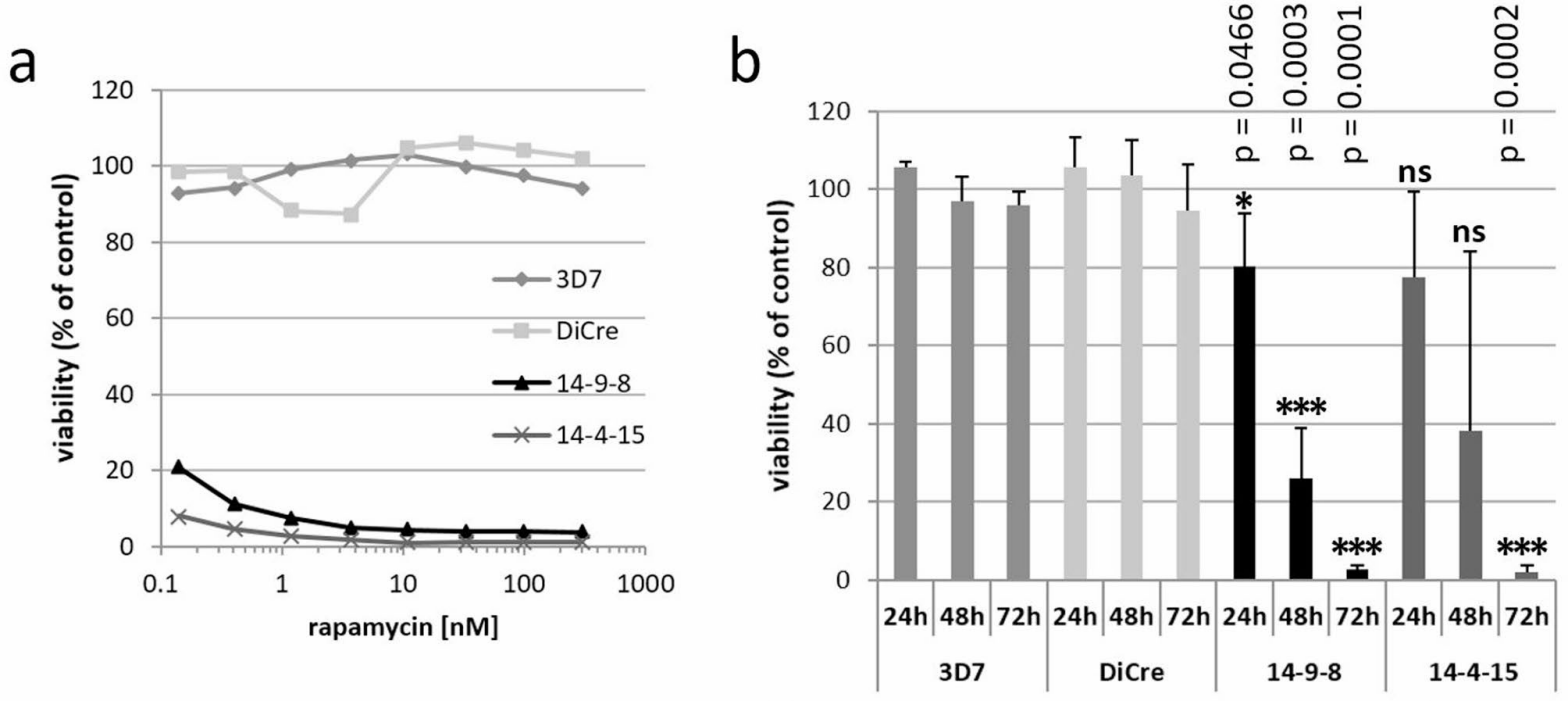
Survival of iPI3K-ko parasites and of the parental strains after rapamycin treatment. (**a**) Parasites of the different lines were treated for 72 h with the indicated concentrations of rapamycin before addition of radioactive hypoxanthine for further 24 h followed by quantification of incorporation of radioactivity into nucleic acids reflecting parasite viability. Incorporation was normalised to the values of untreated control samples and expressed as percentage. Shown is one representative experiment after 72 h rapamycin exposure (*n* = 3). (**b**) Parasites were treated with 100 nM rapamycin for 24 h, 48–72 h before the addition of ^3^H-hypoxanthine for further 24 h. Viability was expressed as percentage of untreated control samples. Data are the mean and SD of 2 to 4 independent experiments. P-values were determined with respect to the corresponding values of the DiCre strain by unpaired *t* test. *p < 0.05, ***p < 0.001, ns: non-significant.

We first analysed the effect of rapamycin induced deletion of the PI3K catalytic domain (hereafter referred to as iPI3K-ko parasites) on parasite viability for which we measured de novo DNA synthesis. Parasite cultures were treated for 72 h with rapamycin followed by the addition of [^3^H] hypoxanthine for 24 h. Parasites were then lysed and the incorporation of radioactivity into DNA quantified. Both iPI3K-ko clones 14-9-8 and 14-4-15 showed very low survival rates of less than 3% and this even at low nanomolar concentrations of rapamycin (Fig. 2a). Importantly, there was no effect on the parental DiCre line 1G5 and the 3D7 strain even at the highest concentration of 300 nM rapamycin indicating absence of toxicity. We also performed these experiments with shorter times of rapamycin treatment of 48 h and 24 h (Fig. 2b). Shorter culture times led to intermediate survival rates, indicating that the viability of the iPI3K-ko parasites was weakly affected early after treatment in the first erythrocytic cycle and that the parasites mainly died in the following cycle. iPI3K-ko clone 14-9-8 was selected for subsequent analyses.

### Specific depletion of PI3P in the PI3-kinase mutant

**Fig. 3:**
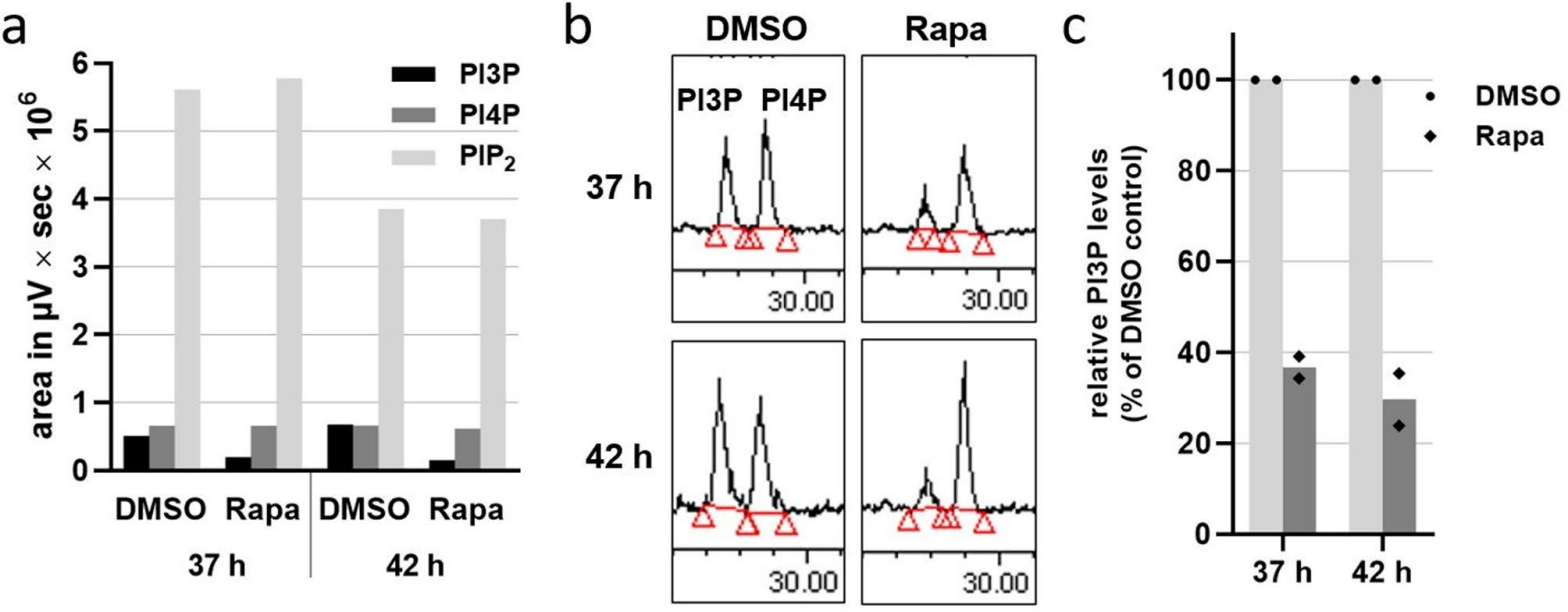
Reduction in PI3P synthesis in the iPI3K-ko mutant. Early ring stage parasites of clone 14-9-8 were treated with 100 nM rapamycin or DMSO as control. After 37 h and 42 h late stage infected cells were separated from uninfected RBCs and purified iRBCs were labelled for 1 h with 100 µCi ^32^P phosphate. Phosphoinositides were extracted, separated by TLC, deacylated, and identified and quantified by HPLC. (**a**) Synthesis of PI3P, PI4P and PI(4,5)P_2_ as defined by the area under the curve of the HPLC chromatogram. (**b**) Chromatograms of the fraction corresponding to the PI-monophosphates between 27 and 30 min. Red lines and triangles delimit the integrated areas. (**c**) Relative PI3P levels in the induced mutant (Rapa) with respect to the non-induced control (DMSO) set to 100%. *n* = 2 biological replicates.

Given that *P. falciparum* PI3-kinase produces PI3-monophosphate^10^ we analysed the phosphoinositide profile in the iPI3K-ko parasites at 37 and 42 h post-invasion (hpi) that also corresponds to the time after addition of rapamycin. While rapamycin treatment had no influence on the amounts of PI4P and PI(4,5)P_2_ at both time points (Fig. 3a), there was a marked reduction in PI3P production, with 37% PI3P remaining after 37 h and 30% remaining after 42 h, both with respect to the control samples (Fig. 3b, c). These results demonstrated that addition of rapamycin to young ring stage parasites of the iPI3K-ko mutant resulted in moderate depletion of PI3P in late stage parasites.

### Growth phenotype of the iPI3K-ko mutant

**Fig. 4:**
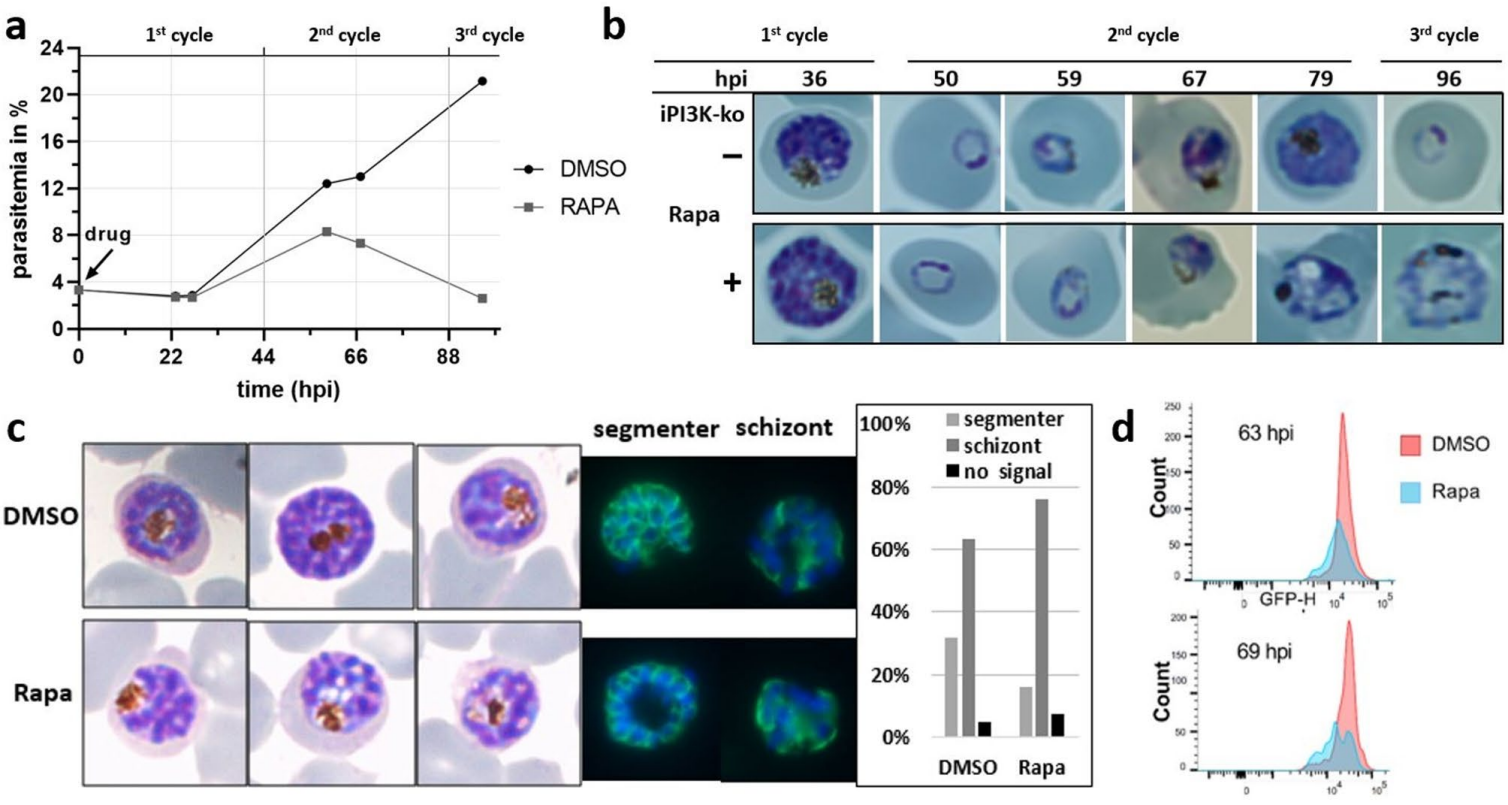
PI3-kinase enzyme is essential for the normal growth and survival of *P. falciparum* during the asexual intraerythrocytic life cycle. (**a**) Growth curve of DMSO and Rapa treated early rings in iPI3K-ko parasites at different time points over a 96 hpi time course. T = 0 corresponds to the time of sorbitol synchronisation and the addition of rapamycin. Of note, the duration of the intraerythrocytic cycle of 3D7 and all derived strains in our conditions is around 44 h. (**b**) Morphology of parasites throughout different developmental stages upon depletion of PI3K. Difco-stained thin film smears illustrate important morphological changes from the trophozoite stage onwards. (**c**) Morphology of iPI3K-ko parasites at the very end of the first cycle. Difco-stained smears of iPI3K-ko parasites at 43 hpi and after addition of rapamycin to 0–4 h ring stages (bottom) or controls (top). On the right immunofluorescence label of MSP1 (green) and DNA (DAPI, blue). Shown is one example of a fully segmented parasite to the left and a schizont on the right. Quantification of the number of fully segmented parasites in control (DMSO) and PI3K-depleted conditions (Rapa). Results of one experiment are shown (*n* = 2). (**d**) Flow cytometry histogram overlay of parasite DNA staining of control and ko parasites at 63 hpi and 69 hpi.

To follow the development of iPI3K-ko parasites rapamycin was added to synchronous ring stage parasites (4 h window) just after synchronisation. The time of synchronisation was defined as t = 0 for all experiments. It is important to note that in our culture conditions the intraerythrocytic cycle of 3D7 and of all derived parasites lines is around 44 h. Compared to mock (DMSO) treated controls, parasitemia was identical during the first cycle and somewhat reduced in the second cycle. PI3-kinase depleted parasites did not pass into the third cycle (Fig. 4a). When we analysed parasite development in more detail we observed that during the first cycle all parasites reached the schizont stage (Fig. 4b, Fig. S2). However, some parasites seemed to be blocked just before the segmenter stage (Fig. 4c) as seen by immuno-staining with anti-MSP1 antisera. More parasites showed a diffuse staining of a schizont instead of the typical “grape-like” pattern seen when daughter merozoites are fully formed. In the second cycle parasites started to appear affected at the trophozoite stage. However, the phenotype was very heterogeneous. Analysis of DNA content by FACS also indicated that iPI3K-ko mutant parasites did not engage normally into DNA synthesis in the second cycle (Fig. 4d, Fig. S2).

### Depletion of PI3K leads to a partial loss of the apicoplast in next generation parasites

**Fig. 5:**
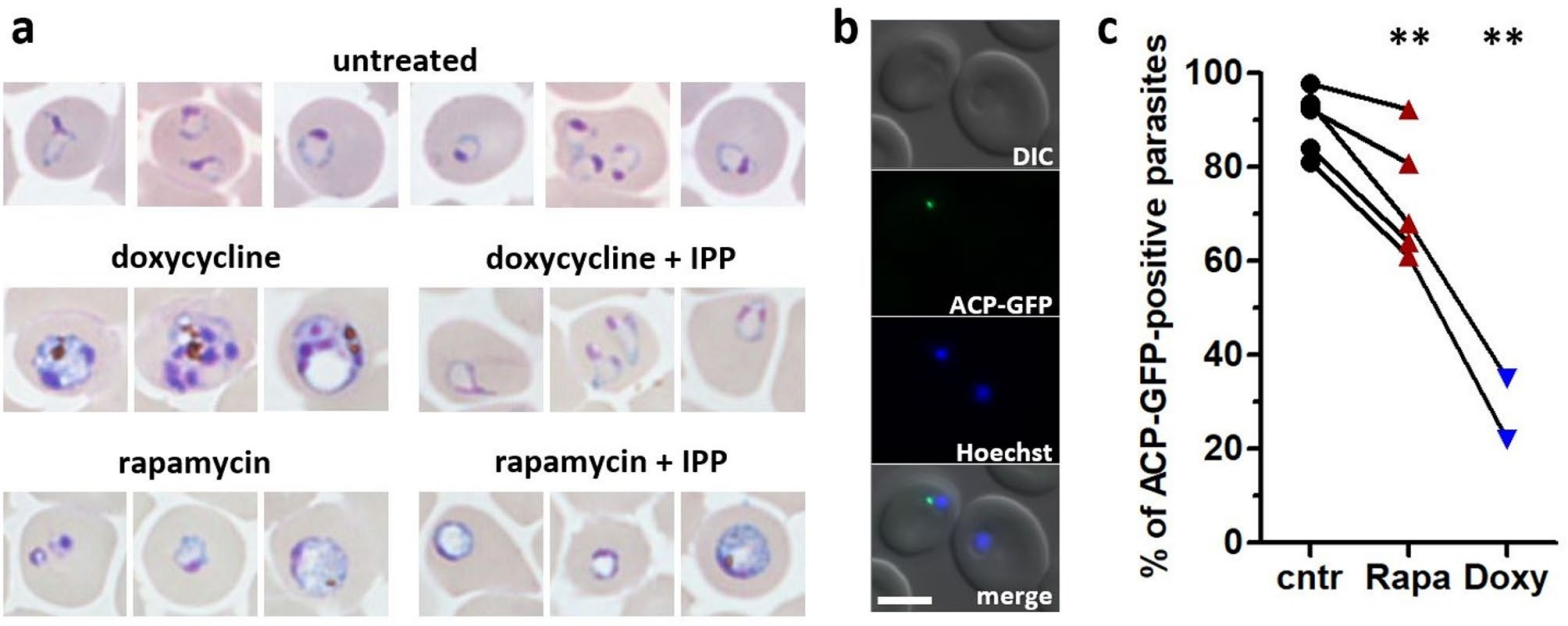
iPI3K-ko parasites exert a minor effect on apicoplast biogenesis in next generation ring stages. (**a**) Parasites at the young ring stage were treated with 2 µM doxycycline, 100 nM rapamycin, and 200 µM IPP. Media containing drugs were changed after 48 h and 72 h. Images were taken 96 h post treatment and three representative images are shown. Only doxycycline + IPP-treated cultures proceeded normally to 3rd generation ring stages. (**b**) Fluorescence microscopy imaging of iPI3K-ko pACP-GFP next generation ring stage parasites at 51 h after rapamycin-treatment. Only one of the two depicted parasites shows apicoplast ACP-GFP labelling. DNA was stained with Hoechst. Zeiss microscopy, 63×, scale bar 5 μm. (**c**) Quantitative analysis of ACP-GFP positive iPI3K-ko parasites in three conditions: DMSO control, 50 nM rapamycin (*p* = 0.0099), or 2 µM doxycycline (*p* = 0.0030), paired two-tailed student *t*-test. *n* = 5 for rapamycin and *n* = 2 for doxycycline. Lines indicate data from the same experiment.

In the related apicomplexan parasite *Toxoplasma gondii* the *Tgpi3k* gene has been shown to be essential for parasite survival through an essential function of PI3P at the apicoplast^15,16^. In *T. gondii* and *P. falciparum* disturbance or loss of the apicoplast through drug treatments or genetic modifications lead to “delayed death”, a phenotype that manifests in the death of the parasite in the cycle following apicoplast interference after invasion of new host cells. Since the observed block of *P. falciparum* development in iPI3K-ko mutants could be an example of delayed death we examined the effect on the apicoplast. The loss of the apicoplast in *P. falciparum* can be induced in vitro by treatment with doxycycline and these parasites can continue to grow in the absence of their apicoplast provided the culture media are supplemented with isopentenyl-pyrophosphate (IPP, a precursor of the mevalonate pathway^40^). When we treated young ring stage cultures of the iPI3K-ko strain with doxycycline with or without IPP, only the IPP supplemented cultures survived as expected and showed ring stage parasites after 96 h (Fig. 5a). However, when the same initial iPI3K-ko parasites were treated with rapamycin with or without IPP, parasites died and none of the cultures showed ring stage parasites after 96 h indicating that delayed death upon loss of the apicoplast was not the (only) reason for parasite death.

We then analysed whether we could observe loss of the apicoplast in the iPI3K-ko mutant. We transfected the strain with an episomal ACP-GFP construct^41^ leading to the expression of GFP in the apicoplast stroma (Fig. 5b). Parasites were analysed at 51 hpi, at the next generation ring stage for the presence of a punctate green fluorescent signal as indicator for the presence of the organelle. We observed that more than 80% of control ring stage parasites showed an apicoplast label and systematically this percentage was lower by an average of 16.5% in the rapamycin-treated parasites in five independent experiments. As expected, the loss of the apicoplast was more drastic in doxycycline-treated parasites (Fig. 5c). These results indicate that there is a functional link between PI3P and apicoplast inheritance in *P. falciparum*. However, the partial loss of the apicoplast is unlikely to be the unique cause of parasite death that occurs in the second cycle.

### PfPI3-kinase is involved in haemoglobin transport by the parasite

**Fig. 6:**
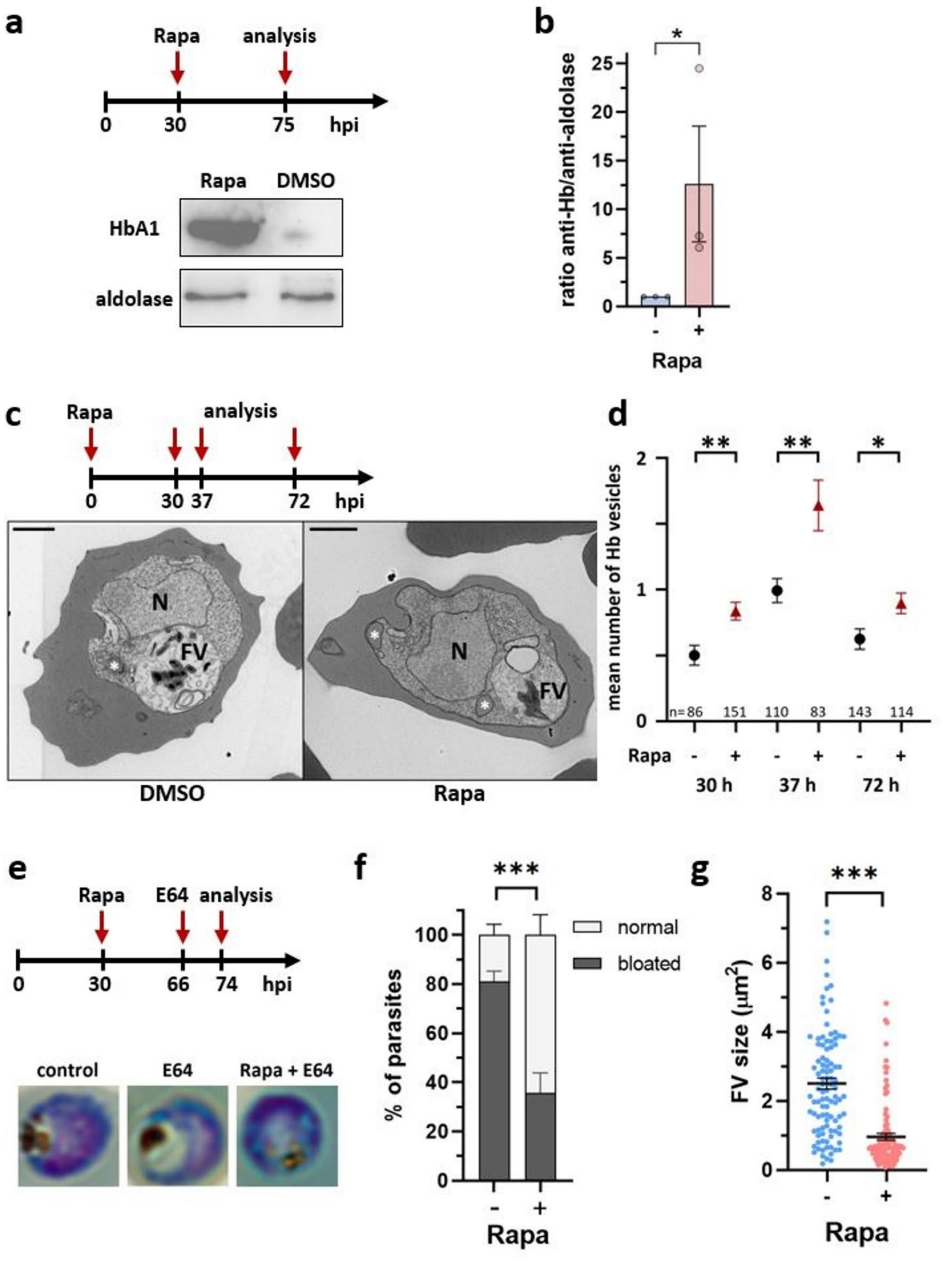
*Pf*PI3-kinase is involved in haemoglobin digestion. (**a**) Schematic representation of the experiment (top) and Western blot analysis (bottom) showing increased levels of Hb in isolated iPI3K-ko parasites treated with rapamycin in comparison to control parasites. Aldolase was used as a loading control. Full-size images of the 3 experiments are shown in Fig. S6. (**b**) The intensity of Hb and aldolase signals from three independent Western blot results were analysed and quantified by Fiji. The ratio of Hb and aldolase of each condition was calculated and normalized (*p* = 0.0371, unpaired *t*-test, *n* = 3 biological replicates). (**c**) Representative electron microscopy images of iPI3K-ko iRBC at 72 hpi post induction with rapamycin in early rings. *: Hb containing vesicle; N: nucleus; FV: food vacuole. Scale bar 1 μm. (**d**) Number of Hb containing vesicles per parasite. Mean ± SEM. *p* = 0.0017 (30 h), *p* = 0.0012 (37 h), *p* = 0.0146 (72 h), unpaired *t*-test. The number (n) of analysed parasites is indicated. (**e**) Schematic representation of the experiment and representative microscopy images of Difco stained smears showing the morphology of untreated parasites, E64 treated control parasites, and E 64 treated parasites after rapamycin treatment of iPI3K-ko. (**f**) Quantification of parasites with bloated and non-bloated FV (*p* < 0.0001, 2way ANOVA). (**g**) Quantification of the food vacuole size upon E64 treatment in 100 parasites of each condition using Fiji analysis software. The bar represents mean ± SEM of five biological replicates; ***p < 0.0001 Mann–Whitney test (*n* = 100 parasites).

Multiple lines of evidence suggest that PI3P plays a crucial role in the normal functioning of the food vacuole and in Hb digestion in *Plasmodium* parasites^10,17,22,35,42^. We thus used our iPI3K-ko mutant to analyse the effect of PI3P depletion on Hb digestion in the trophozoites of the second generation. To do so, rapamycin was added at 30 hpi, i.e. to synchronised trophozoites of the first cycle (Fig. 6a). Under these conditions, development of rapamycin-treated parasites was blocked mainly at the schizont stage of the second cycle (Fig. S3). Parasites at 75 hpi (trophozoites, 30 h after reinvasion) were isolated from the host cells by saponin treatment and the undigested Hb within the parasite was quantified using a commercial antibody against Hb. By Western blot we observed an important accumulation of about 12.6-fold in parasites depleted for PI3-kinase (Fig. 6a, b). Hb-containing vesicles were quantified in electron microscopy images of infected RBCs. PI3-kinase depletion led to an increase in Hb-containing vesicles further asserting our findings (Fig. 6c, d). We verified that these vesicles were not mistaken as lipid droplets that have been described to form physical interactions with the food vacuole^43^ and found no change in the number or the size of lipid droplets (Fig. S4). In summary, our results indicate that upon PI3-kinase depletion, Hb digestion is affected with reduced transport to the food vacuole and larger amounts of undigested Hb being retained in vesicles following Hb uptake.

To analyse food vacuole function in the mutant, we next blocked food vacuole Hb digestion by treatment with the cysteine-protease inhibitor E64^44^ which leads to the development of a bloated food vacuole in control parasites. We induced the iPI3K-ko mutant at the trophozoite stage and blocked food vacuole digestion in young trophozoites of the next generation. We analysed the morphology of the food vacuole 8 h later (Fig. 6e). In control parasites treated with E64, the bloated phenotype was observed in 81% of parasites. In contrast, in the absence of PI3-kinase (Rapa) only 36% of E64-treated parasites displayed bloated food vacuoles (Fig. 6f). In addition, the mean size of bloated food vacuoles was found to be 2.5-fold bigger in the control parasites (Fig. 6g). In conclusion, our data suggest that the absence of PI3-kinase enzyme inhibits the delivery of Hb vesicles to the parasite food vacuole, leading to accumulation of undigested Hb in the parasite cytosol.

### Depletion of PI3K leads to a partial reversal of artemisinin resistance in a K13 mutant background

**Fig. 7:**
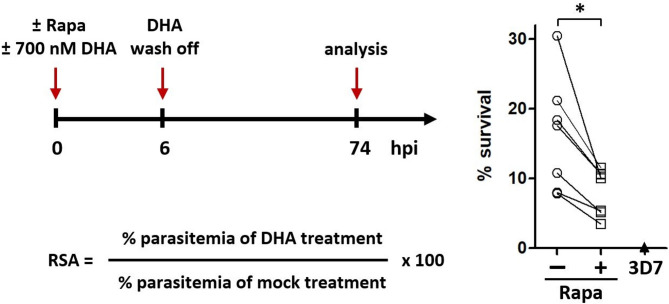
Decreased level of artemisinin resistance in iPI3K-ko/K13-C580Y double mutant parasites. Synchronous very early-ring-stage parasites of 3D7 and iPI3K-ko/K13-C580Y parasites were treated with or without rapamycin and with or without 700 nM of dihydroartemisinin (DHA) for 6 h before wash off of DHA (with continued treatment with rapamycin). The RSA values were calculated as shown in seven independent experiments for iPI3K-ko/K13-C580Y parasites and 5 experiments for the DHA-sensitive strain 3D7. *p = 0.0156 paired two-tailed *t*-test (Wilcoxon).

Resistance to artemisinin is dependent on mutations in *Pf*K13 and it has been shown that increased levels of the lipid PI3P correlate with increased resistance^24^. In order to study the link between PI3-kinase/PI3P lipid abundance and artemisinin resistance, we introduced the K13-C580Y mutation by CRISPR-Cas9 using previously described constructs in the iPI3K-ko strain^45^. The resulting parasites, iPI3K-ko/K13-C580Y, were then used in standard ring stage survival assays to quantify the level of resistance to DHA^46^. As expected, in the absence of rapamycin the strain was found to be resistant to DHA in contrast to the sensitive 3D7 strain (Fig. 7). In seven independent assays we observed that upon addition of rapamycin the iPI3K-ko/K13-C580Y mutant was less resistant to DHA than the corresponding mock-treated parasites. It should be noted that the experimental setup probes the effect of PI3-kinase loss after the DHA pulse. Our observations indicate that lower levels of PI3-kinase and PI3P lipid are linked to reduced artemisinin resistance and thus support the initial findings^24^ that higher levels of PI3-kinase and PI3P lipid are predictive of higher levels of artemisinin resistance.

## Discussion

Studying genes that encode proteins with essential functions during the erythrocytic cycle of *P. falciparum* remains challenging although different techniques are now available to regulate targets at the DNA, RNA and protein level^39,47–52^. We chose the *loxP*/DiCre conditional deletion^39^ of the *pi3k* gene (or more precisely of the catalytic domain of the *pi3k* gene) because we expected a clean phenotype as a consequence of definitive removal of the gene in the individual parasite. Indeed, excision was effective and strictly dependent on induction by rapamycin (Fig. 1d, e). We found that *Pf*PI3-kinase is an essential enzyme in *P. falciparum*. This has previous been indicated by unsuccessful targeted deletion of the *pi3k* gene^17^ and the negative fitness score of piggyBac saturation mutagenesis^53^ while a knock down approach using the TetR/DOZY system did not show a phenotype^54^. We consider that in our mutant DiCre recombination was close to complete since 72 h after the addition of rapamycin less than 3% of iPI3K-ko parasites were still capable of de novo synthesis of DNA (Fig. 2b). We also validated that the product of PI3-kinase enzyme, PI3P lipid synthesis, was specifically reduced in the iPI3K-ko mutant (Fig. 3c). At the same time the lipid analysis also revealed that PI3P was still produced although at low levels indicating that some PI3-kinase enzyme activity was still present around 40 h after the addition of rapamycin. This latter finding likely explains why the majority of iPI3K-ko parasites were still capable to pass to the second cycle even though rapamycin had been added to very young ring stage parasites, and recombination of the locus had occurred during the first 24 h.

The fact that iPI3K-ko parasites mainly died at the trophozoite stage of the second cycle is reminiscent of a delayed-death phenotype that occurs as a consequence of a defective apicoplast^55^. An essential function of PI3P/PI3-kinase at the apicoplast has been shown in the related apicomplexan parasite *T. gondii*, where interfering with PI3P lipid or with PI3-kinase leads to apicoplast loss and parasite death^15,16^. In *P. falciparum* the link is more indirect. A fluorescent PI3P-sensor expressed by the parasite mainly accumulated around the food vacuole but also labelled the apicoplast^10^. Surprisingly, certain autophagy-related (Atg) proteins have been linked to the apicoplast in *Plasmodium*. Autophagy generally is a cell survival process involving recycling of part of the cytoplasm through degradation and importantly, autophagy is a PI3P-dependent process^13^. The *Plasmodium* genome encodes only a limited set of Atg protein^56^ that have been suggested to have non-canonical functions. It is less clear whether autophagy performs degradative functions in erythrocyte stages. Atg8 and Atg18 localise to the apicoplast in *P. falciparum*^57,58^ (but also to vesicles and the food vacuole, see below) and conditional mutants of Atg8 and Atg18 in *P. falciparum* and *T. gondii* show apicoplast loss and parasite death in both species^57,59,60^. Consequently, it has been suggested that one essential function of the Atg proteins in Apicomplexa involves trafficking to the apicoplast^61^.

To study the effect of PI3-kinase disruption on the apicoplast we introduced a fluorescent apicoplast marker in our iPI3K-ko mutant. We indeed observed that some parasites lose their fluorescent apicoplast label in second generation ring stages and this likely reflects loss of the organelle in these parasites (Fig. 5). This demonstrates that PI3-kinase also has an important function at the apicoplast in *P. falciparum* and we hypothesise that PI3P at the apicoplast might be at the origin of the recruitment of Atg proteins to the organelle. Isoprenoid synthesis is the sole metabolic activity of the apicoplast that is essential during the blood stage^21^. And in culture the apicoplast can be dispensable in *P. falciparum* blood stages if IPP is added to the medium^40^. The iPI3K-ko mutant, however, could not be rescued upon simultaneous addition of IPP and rapamycin treatment. This demonstrates that loss of apicoplast function was not the only reason for parasite death and argues against a delayed death phenotype in this mutant. It is thus more likely that the iPI3K-ko parasites predominantly die in the second cycle because some PI3-kinase activity is still present at the end of the first cycle (Fig. 3), sufficient to support passage to the next cycle, before activity becomes progressively limited leading to parasite death mainly at the trophozoite stage.

Our results on the phenotype of the iPI3K-ko mutant identified a defect in Hb uptake and/or Hb digestion in the parasite food vacuole. We found substantial amounts of undigested Hb inside isolated parasites (i.e. separated from the Hb-containing RBC host cell) (Fig. 6a). This is not the case in control parasites because within the parasite Hb is rapidly digested in the food vacuole. This finding was confirmed by electron microscopy imaging where we observed an increase of Hb-containing structures within the parasites (Fig. 6c). Taken together these observations suggest that in the absence of PI3P lipid Hb uptake by the parasite can still be initiated leading to the formation of Hb vesicles in the parasite likely because Hb transport to the food vacuole is affected. A similar effect has previously been described when high concentrations of the PI3-kinase inhibitors wortmannin and LY294002 were used^22^. Our results obtained with the bloated food vacuole assay^44^ further corroborate these findings. A strong reduction in the number and in the size of bloated food vacuoles was observed in the iPI3K-ko mutant upon treatment with a cysteine protease inhibitor that blocks Hb digestion (Fig. 6f). There is less bloating of vacuoles in the iPI3K-ko mutant likely because there is less undigested Hb present in the vacuole as a consequence of a defect in Hb delivery.

Our observations with the iPI3K-ko mutant show certain similarities with the phenotype of a conditional *Pf*Vps45 mutant^35^. In other organisms Vps45 is involved in endolysosomal transport^62,63^. In *P. falciparum* a knock-sideways mutant of *Pf*Vps45 revealed that the protein is an essential factor in the uptake of host cell cytoplasm and in the mutant, vesicles accumulate within the parasite and are not delivered to the food vacuole. Interestingly, a proportion of these vesicles was positive for PI3P^35^. A recent publication identified Rabenosyn-5 (Rbsn5L) and Rab5b as partners of Vps45 in this process and conditional inactivation of these proteins resulted in a similar phenotype of Hb vesicle accumulation^32^. These vesicles also contain PI3P and thus present characteristic features of early endosomal membranes^32,35^. However, direct binding of *Pf*Rbsn5L to PI3P could not be detected although the proteins contains a FYVE domain, which is one of the known PI3P-binding domains. Other PI3P-binding proteins have nevertheless been identified at the food vacuole or at Hb-containing vesicles. One example is *Pf*PX1, identified in a genomic screen for phosphoinositide-binding proteins, that when absent displayed increased accumulation of cytosolic Hb^17^. Another example is the autophagy-related protein *Pf*Atg18 that binds PI3P and has also been localised to the food vacuole and to Hb-containing vesicles^64,65^. It is thus tempting to speculate that the presence of PI3P on Hb-containing vesicles might be necessary for the recruitment of PI3P-binding proteins like PX1, Atg18 and the Vps45 complex and for successful delivery of Hb containing vesicles to the food vacuole.

The antimalarial activity of artemisinin is dependent on cleavage of their endoperoxide bridge thereby producing free radicals that alkylate indiscriminately proteins throughout the parasite^66,67^—a situation that may be described as a proteopathy. Parasite resistance to artemisinin has been linked to parasite Hb digestion because it liberates reactive haem which activates artemisinin for its antimalarial activity^7,31,37,68^. The hypothesis is that resistance is conferred by a temporary reduction of Hb degradation in young ring-stages, resulting in lower levels of free haem and reduced activation of artemisinin allowing parasites to survive the peak levels of the short-lived artemisinin. Following this line of reasoning, in the iPI3K-ko/K13-C580Y double mutant one could have expected an increase in artemisinin resistance, as we have clearly shown that PI3P depletion leads to reduced Hb catabolism. However, we did in fact observe the opposite, a reduction in resistance. This is likely explained by a difference in timing. K13-mutations reduce Hb digestion at the time of artemisinin treatment of young ring stages while in the iPI3K-ko mutant effective PI3P depletion only takes place later towards the schizont stage and Hb digestion is affected in the second cycle. Thus, the effect of lower PI3P leading to reduced resistance in our study is probably not related to the reduction in Hb catabolism, but to additional mechanisms that take place after artemisinin treatment.

It has previously been shown that high PI3P levels correlate with artemisinin resistance in both clinical isolates and K13-mutated transgenic laboratory lines^24^. The Kelch domains of K13 are suggested to be implicated in ubiquitination of target proteins and their proteasomal degradation, *Pf*PI3-kinase being one of the targets. The K13-C580Y mutation was suggested to increase the protein level of PI3-kinase via a decrease in its ubiquitination, thereby leading to higher PI3P levels^24,28^. More cellular PI3P might then improve recovery of artemisinin-induced proteopathy through enhanced cellular repair pathways like the unfolded protein response and autophagy-like processes^28^. Concordant with this hypothesis, Atg18-positive autophagic vesicles have been observed in K13-mutant strains^38,69^. Atg18 binds PI3P^57^ and an Atg18 T38I polymorphism associates with artemisinin resistance and confers enhanced parasite survival under nutrient deprivation^70^. It is therefore plausible that the reduced amounts of PI3P in the iPI3K-ko mutant dampens repair mechanisms like autophagic processes that might associate with K13-C580Y, leading to higher proteotoxic stress and to an overall increase in artemisinin susceptibility.

In summary, generating a conditional PI3-kinase mutant showed that this enzyme is essential for *P. falciparum* blood stages with functions in apicoplast biology and host cell Hb digestion. The observed higher sensitivity to artemisinin when PI3P levels are low likely points towards the importance of PI3P-dependent cellular repair processes in the mechanism of resistance. All in all, we provide the tool to decipher the multiple roles of PI3-kinase in the malaria parasite in more detail in future studies.

## Methods

### Parasite culture

Human blood obtained as donations from anonymized individuals was provided by the local blood bank (Etablissement Français du Sang, Pyrénées Méditerranée, France) under the approval number 21PLER2016-0103. *P. falciparum* strains were cultured in O^+^ or A^+^ human RBCs, in complete medium composed of RPMI 1640 with 2 mM L-glutamine and 25 mM Hepes, supplemented with 100 µM hypoxanthine, 0.5% albumax II and 10 µg/ml gentamycin. Parasite cultures were maintained at 37 °C in an atmosphere of 5% O_2_, 5% CO_2_, 90% N_2_ either in static or shaking conditions. In our culture conditions the intraerythrocytic cycle of 3D7 and of all derived parasites lines is around 44 h. Synchronization of cultures was achieved by a sequential recovery of late stage parasites by density centrifugation using 64% Percoll/RPMI, subsequent culture with fresh RBCs to allow invasion, followed by selected lysis of remaining late stage parasites by 5% D-sorbitol to obtain an early ring stage culture (< 4 h post invasion, hpi). Parasitemia was monitored by Difco staining of thin film smears or by FACS. Drugs used for selection were WR99210 at 2.5 nM (Jacobus Pharmaceuticals), blasticidin at 2.5 µg/ml (Sigma), and DSM1 at 1 µg/ml. Rapamycin (LC Laboratories) was used at 100 nM or 50 nM during assays.

### Generation of transgenic parasite lines with LoxP sites integrated at the PI3-kinase locus

The *Pf*PI3K coding sequence is 6399 bp long without intron. The catalytic domain encompasses about 265 aa and is located at the extreme C-terminal end of the protein^10^. Our published result showed that adding a C-terminal tag had not been possible in *P. berghei*, suggested that removal of the catalytic domain should be sufficient to block PI3-kinase activity. Careful examination of sequence alignments and secondary structure predictions of PI3-kinase proteins from different *Plasmodium* species identified a poorly conserved and unstructured sequence of about 150 amino acids upstream of the catalytic domain that contains repeat motives in several *Plasmodium* species. We selected two positions for which we expected that an insertion of 12 amino acids encoded by a *loxP* site would not interfere with enzyme function (Fig. S1). Of note, the generation of these constructs had been initiated before the design of the genetic module loxPint that carries a *loxP* site in a short synthetic intron^71^.

The second *loxP* site necessary for Cre mediated removal of the 3′ end of the *Pfpi3k*gene was present in the plasmid backbone downstream of the hDHFR cassette. The transfection plasmid pHH1-lox2-iPI3K-ko used to target the *Pfpi3k* locus by single homologous recombination is based on plasmid pHH1_SERA5del3DC^39^ and contained the following elements (Fig. 1a): (1) a 1.2 kb sequence of the extreme 3′ end of the *Pfpi3k* gene including one *loxP* site at two possible positions in the beginning of this sequence; this synthetic sequence encoding the catalytic domain had been re-codonised to avoid recombination with the *pi3k*-locus; (2) a homology region of 1.1 kb of the immediate upstream PI3K coding sequence that will serve for integration into the genome by single homologous recombination; (3) a heterologous 3′UTR (of PbDHFR/TS) for transcriptional termination prior to Cre-mediated recombination; (4) an hDHFR expression cassette conferring resistance to WR22910 followed by the second *loxP* site in the same orientation as the first one; and (5) the 3′UTR of the *Pfhsp86* gene that upon Cre-mediated recombination will replace the catalytic domain and assure transcriptional termination of the truncated gene. The two plasmids pPI3K-lox1and pPI3K-lox2 varied only in the position of the loxP site upstream of the PI3K catalytic domain. The recipient parasite line was the 1G5 line, a 3D7 derived parasite that carries the DiCre expression cassette downstream of the SERA5 gene^39^. Ring stage parasites at 5% parasitemia were transfected by electroporation with 100 µg plasmid, and selected with WR99210 at 2.5 nM. Transgenic parasites were obtained with both constructs. The populations were subjected to multiple rounds of WR22910 drug cycling to enrich in parasites that had integrated the transfection construct into the *pi3k* locus and cloned by limiting dilution. One clone each was obtained for the two independent transfections of construct pPI3K-lox2, and named PF14-9-8 and PF14-4-15.

For the analysis of apicoplast inheritance the iPI3K-ko line was transfected with plasmid pLN-ACP-GFP that encodes the green fluorescent protein fused to the ACP leader peptide resulting in translocation of GFP into the apicoplast.

### Generation of a double transgenic parasite line iPI3K-ko and Kelch13 C580Y

To generate an artemisinin-resistant line of our iPI3K-ko mutant we introduced a traceless C580Y mutation of the *kelch13* gene in our iPI3K-ko by CRISPR-Cas9. For this our iPI3K-ko parasite line was transfected with the plasmids pUF1-Cas9 and pL7-k13-g16 as previously described^45^. Transgenic parasites were selected with DSM1 and cloned by limiting dilution. The genotype was screened by PCR using specific primer pairs: p853/p854 for wild type *kelch13*, and p852 / p854 for *kelch13* C580Y. One mutant clone was obtained and the expected editing of the *k13* locus confirmed by amplifying the locus with p854 and p855 and sequencing the amplicons with p856.

### Quantitative PCR on genomic DNA

Genomic DNA was extracted using the Qiamp blood kit (Qiagen). Two primer pairs were designed that amplify across the *loxP* sites (coloured arrows in Fig. 1c). One pair amplifies from the *pi3k*-integration sequence and the optimized sequence and thus represents the genomic situation before Cre recombination (termed the integrated gene). The second pair amplifies from the integration sequence and the 3′UTR of the *hsp86* gene, a primer combination that can only amplify after successful Cre recombination (termed excised gene). For amplification of a reference gene we chose primers in the 5′ part of the *pi3k* gene upstream of the sequences used for the transfection construct. qPCR reactions were performed on a Roche LightCycler 480 instrument using SYBR Green I Master reagent and data were analysed by LC480 software version 1.5. The ratio of Cp values to the reference gene were calculated and presented at log2 scale.

### Measurement of parasite survival

We applied a method based on the Desjardin assay^72^ to quantify parasite survival upon addition of rapamycin. Serial 1:3 dilutions of rapamycin starting at 300 nM final concentration were prepared in 96 well plates and iPI3K-ko parasites added at 0.5% parasitemia and 1.5% haematocrit. Plates were incubated for either 24 h, 48–72 h before the addition of 30 µL medium containing 0.5 µCi of ^3^H-hypoxanthine for additional 24 h and then frozen. Cells were lysed by thawing and the parasite nucleic acids were collected on glass-fiber filter plates using a FilterMate cell harvester (Packard Instruments). The radioactivity was counted on a Microbeta2 counter (Packard Instruments). Background was obtained from non-infected erythrocytes and the value was subtracted. Parasitemia were evaluated and expressed as percentage of the control (without drug).

### Phosphoinositide quantification

Highly synchronized ring stage parasites 5 h after invasion were split in 4 cultures, two of which were treated with 100 nM rapamycin while the other two received DMSO mock treatment. At 37 h and 42 h post-invasion iRBC were purified by VarioMACS from both cultures and labelled with ^32^P phosphate for 1.5 h. Lipids were then extracted and separated by thin layer chromatography. The zone corresponding to PI-mono- and bis-phosphates combined was recovered and lipids were de-acylated before analysis by HPLC as described previously^10^.

### Western blotting

*P. falciparum* iPI3K-ko parasites were synchronized and the culture started at 1% parasitemia and 5% haematocrit. Rapamycin was added at 30 hpi, and parasites of each condition (DMSO and Rapamycin) were later collected at 75 hpi. Parasites were pelleted and washed with PBS, then treated with 0.015% saponin at 4 °C for 15 min to allow RBC lysis. After RBC lysis, parasites were centrifuged at 1800*g* for 5 min. The process was repeated three times until the supernatant becomes colourless. The pelleted parasites were washed in cold PBS, followed by incubation in 2× Laemmli lysis buffer. Parasite proteins were harvested by centrifugation at 20,000*g* for 30 min. The proteins were transferred onto a nitrocellulose membrane by the Trans-Blot Turbo Transfer system (Bio-Rad). The membrane was then incubated with blocking buffer (5% skimmed milk in TBS), followed by incubation with monoclonal mouse anti-HBA1 (clone 4F9, Sigma, WH0003039M2) primary antibodies (1/1000 dilution) at 4 °C overnight. The membrane was then washed three times with TBST (TBS containing 0.2% Tween20), and incubated for 1 h with secondary antibodies goat anti-Mouse IgG-HRP (Promega W4021) at 1/5000 dilution in blocking buffer. After the final washes, the signal was developed using the clarity Western ECL substrate (Bio-Rad). Membranes were then washed before identical treatment with mouse anti-aldolase (Abcam, AB 207.494). The aldolase band intensities were used as a loading control for samples in all Western blots. Quantification of bands intensity was done using ImageJ analysis, where HBA1 signals were normalized to the corresponding aldolase signals.

### Immunofluorescence assay

For MSP1 staining, thin blood smears were prepared 43 hpi and slides were frozen. After thawing, areas of interest were delimited with Immunopen. Cells were fixed with 4% paraformaldehyde (PFA) in phosphate-buffered saline (PBS) for 30 min and permeabilised with 0.1% Triton X-100/PBS for 10 min followed by 2 washes for 5 min in PBS. Samples were blocked with 3% BSA/PBS for 1 h and washed again twice for 5 min in PBS. Mouse anti-MSP1 19 kDa antibody (gift of M. Blackman) was used at 1/1000 dilution in 3% BSA/PBS and incubated for 45 min at 37 °C in a closed humidified chamber. After 2 washes with PBS samples were incubated with antibody Alexa488 anti-mouse at 1/1000 dilution in 3% BSA/PBS 30 min at 37 °C. After two washes with PBS 4 µL of vectashield with dapi were added and samples sealed with coverslips and nail varnish. Acquisitions were done on a Zeiss Axio upright microscope and Zen software.

### Electron microscopy

Highly synchronized *P. falciparum* cultures (0–3 h) were treated with 100 nM rapamycin or DMSO. Sample were fixed by adding 25% glutaraldehyde EM grade directly in the culture medium in order to obtain a final concentration of 2.5%. After 10 min at room temperature, cells were centrifuged and the pellet resuspended in 20 pellet volumes of cacodylate buffer 0.1 M containing 2.5% glutaraldehyde and 5 mM CaCl_2_.The suspension was left 2 h at room temperature and then kept at 4 °C in the fixative until further processing. All the following incubation steps were performed in suspension, followed by centrifugation using a benchtop microcentrifuge. Cells were washed with cacodylate buffer and post-fixed with 1% O_s_O_4_ immediately followed by 1.5% potassium ferricyanide in the same buffer. After washing with distilled water, samples were incubated overnight in 2% uranyl acetate in water, washed with water, incubated in Lead aspartate and washed again and dehydrated in graded series of acetonitrile. Impregnation in Epon 812 was performed in suspension in Epon: acetonitrile 50:50 and two times 1 h in 100% Epon. After the last step, cells were pelleted in Fresh epon and polymerized 48 h at 60 °C. All incubation and washing steps were performed using a microwave processor PELCO Biowave Pro+ (TED PELLA) except for the overnight incubation. 100 nm sections were made with an ultramicrotome Leica UCT collected on silicon wafers and observed on a Zeiss Gemini 360 scanning electron microscope on the MRI EM4Bio platform under high vacuum at 2 kV. Final images were acquired using the Volutome backscattered electron detector (Zeiss), a process that results is TEM-like images. The working distance was between 3.5 and 4 mm and the pixel size 5 nm. Independent random tiles were automatically using Atlas 5 software. Vesicular structures with a density similar to the cytoplasm of the surrounding RBC were counted as Hb vesicles (*n* = 80 to 160). Representative pictures were acquired with a pixel size of 1 nm for illustration.

### Labelling the neutral lipids of *P*. *falciparum* with nile red

iPI3K-ko parasites were synchronized by Percoll/sorbitol synchronisation and rapamycin was added 30 hpi. Parasites of each condition (DMSO and Rapamycin) were stained at 75 hpi (corresponding to 31 h in the second cycle) with Nile Red at a final concentration of 1 µg/ml for 20 min at 37 °C followed by 10 min incubation on ice. Samples were then washed three times with PBS, before analysis by spinning disc CSU-W1 microscopy at 100× objective, with an excitation wavelength of 515 nm and 585 nm emission.

### Flow cytometry analysis

To follow parasite DNA synthesis during experiments, 5 µL of packed cell volume (PCV) of parasite cultures were fixed with a solution of 4% paraformaldehyde (PFA) with 0.025% glutaraldehyde in PBS, incubated for 2 h at RT, and then stored at 4 °C. Two µL of the previously fixed PCV were used, quenched with 0.1 M glycine for 30 min and washed thrice with PBS, before staining with 5× SYBR green fluorescent dye for 30 min of at 37 °C. FACS data acquisition was performed directly thereafter on a BD FACSVerse 8 colour cytometer equipped with standard filters. Initial gating was performed on stained, uninfected RBCs to identify infected RBC. FACS analysis was done using FlowJo software.

### Ring-stage survival assay (RSA)

RSA were performed following the standard protocol^46^. Tightly synchronized early ring parasites (0–3 hpi) were exposed to a 6 h treatment of 700 nM DHA (Sigma) or DMSO, in the presence or absence of 50 nM rapamycin. Then parasites were washed thrice and returned to culture for 66 h. Blood smears were prepared and stained with Difco. Each condition was done in duplicates and 15,000 erythrocytes were counted by light microscopy for viable ring stage parasites. Parasite survival rate (%RSA) was expressed as a percentage by dividing the number of viable parasites between the DHA-treated and untreated samples. Seven independent experiments were performed.

### Statistical analysis

Data analysis and plots were generated using Excel (Microsoft) and Prism (GraphPad). Statistical significance was determined by non-parametric Mann–Whitney test for *n* > 3, or by unpaired two-tailed *t*-test (GraphPad Prism). Means associated with the standard error of the mean (SEM) are illustrated on the figures.

## Electronic supplementary material

Below is the link to the electronic supplementary material.

Supplementary Material 1

## Data Availability

All data generated or analysed during this study are included in this published article (and its Supplementary Information files).

## Abbreviations

DHA: Dihydroartemisinin
Hb: Haemoglobin
hpi: Hours post-invasion
IPP: Isopentyl pyrophosphate
PBS: Phosphate-buffered saline
PI: Phosphatidylinositol
PI3P: Phosphatidylinositol 3-phosphate
RBC: Red blood cell
iRBC: Infected red blood cell
