# Supplementary material for: PI3-kinase has multiple functions in asexual blood stages of Plasmodium falciparum
Source: Sci Rep. 2025 May 14;15:16762. doi: 10.1038/s41598-025-01397-1 (PMC12078608; doi:10.1038/s41598-025-01397-1)
Supplement: Supplementary file 1 — Supplementary Material 1 [file 41598_2025_1397_MOESM1_ESM.pdf]

Supplementary Information

PI3-kinase has multiple functions in asexual blood stages of *Plasmodium falciparum*

Reem Al Monla, Maria Penzo, Alice Vallentin, Rakhee Lohia, Jeremy Vincent, Laurence Berry, Ana Rita Gomes, Rachel Cerdan and Kai Wengelnik

Supplemental Figures S1 – S6

a

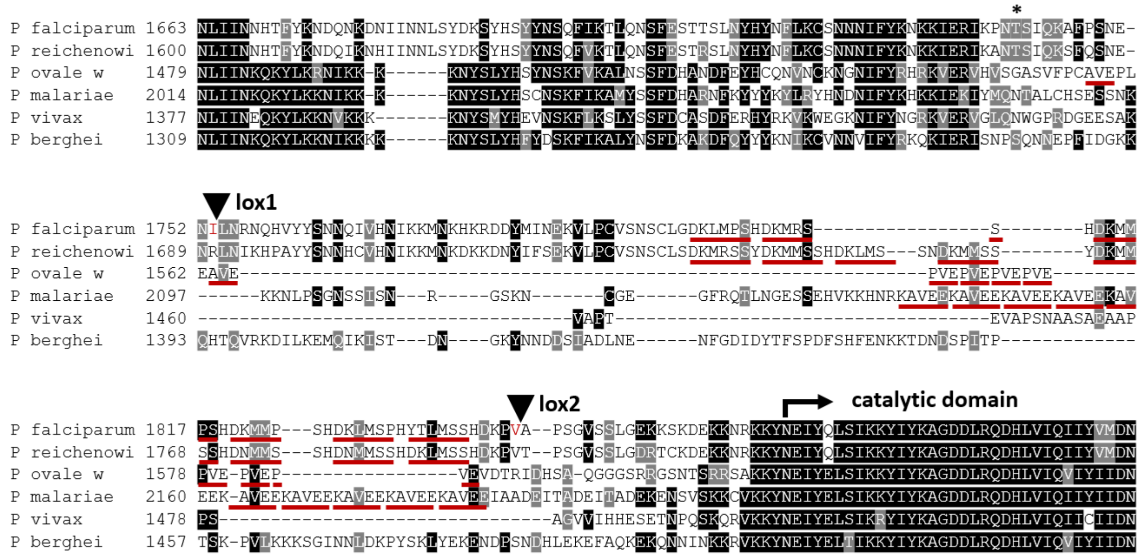

b **ATAACTTCGTATA**ATGTATGC**TATACGAAGTTAT**cc

I T S Y S I H Y T K L S

Supplemental Figure S1: Sites of insertion of *loxP* sequences in the *P. falciparum* PI3-kinase coding sequence. **(a)** Multiple sequence alignment of PI3-kinase proteins of human, primate and rodent malaria species. Only the region immediately upstream of the catalytic domain (indicated by an arrow) is shown. The black triangles indicate the two positions after the amino acid labelled in red that had been chosen in the two transfection constructs to insert a *loxP* site in frame with the coding sequence. In *P. falciparum* and other species this region contains sequence repeats (underlined in red). The two clones 14-9-8 and 14-4-15 have the *loxP* site inserted at position lox2. The asterisk labels Thr1741 and corresponds to the beginning of the re-codonised sequence. Sequences are from reference strains from PlasmoDB: *P. falciparum* 3D7, *P. reichenowi* CDC, *P. ovale wallikeri*, *P. malariae* UG01, *P. vivax* P01, *P. berghei* ANKA. The alignment has been generated with ClustalOmega and coloured with BoxShade. **(b)** Two bases were added to the 34 bp *loxP* site (capital letters) to allow translation (shown below) and read through without inducing a frame shift or a stop codon. This sequence was inserted at the two positions indicated above.

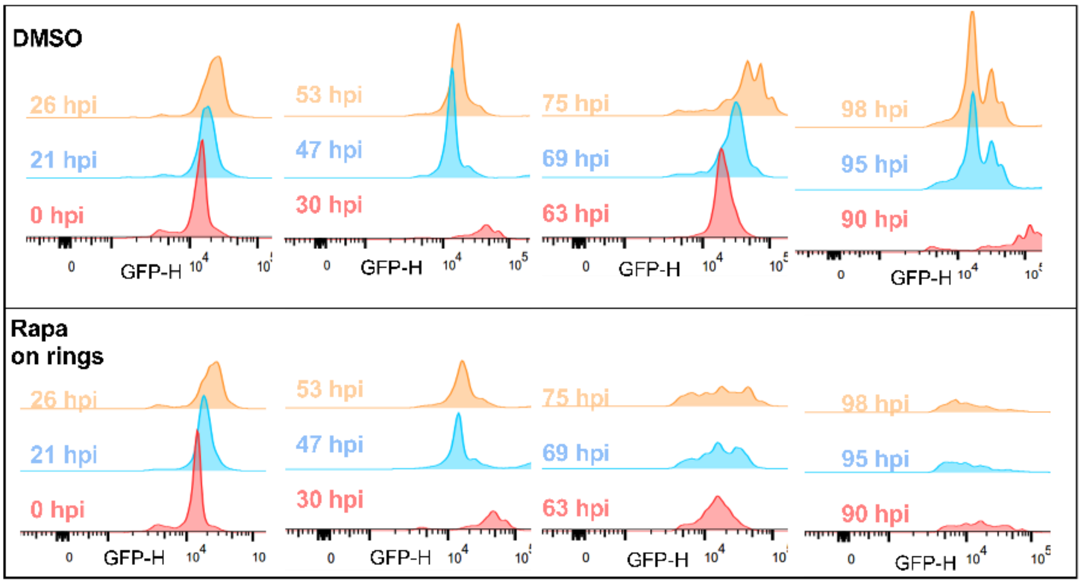

Supplemental Figure S2. Flow cytometry analysis of iPI3K-ko parasite DNA content after treatment at the young ring stage. The SYBR-green fluorescence profile was similar for control (DMSO) and rapamycin (Rapa) treated cultures up to 63 hpi (corresponding to 19 h in the second cycle) even if Rapa cultures had lower parasitemia in the second cycle (from 47 hpi). At the transition to trophozoites only a fraction of rapamycin treated parasites synthesised DNA. Only control parasites pass into the 3rd cycle (after 90 hpi). Shown are the histograms of one representative experiment (n = 3).

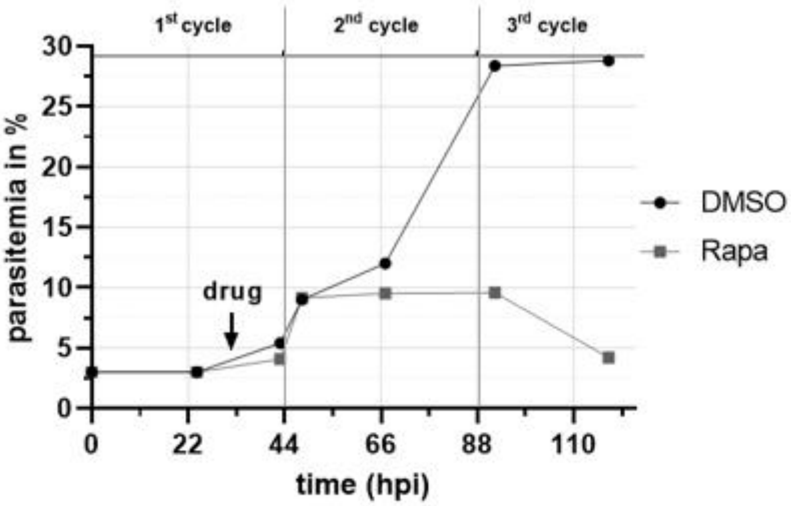

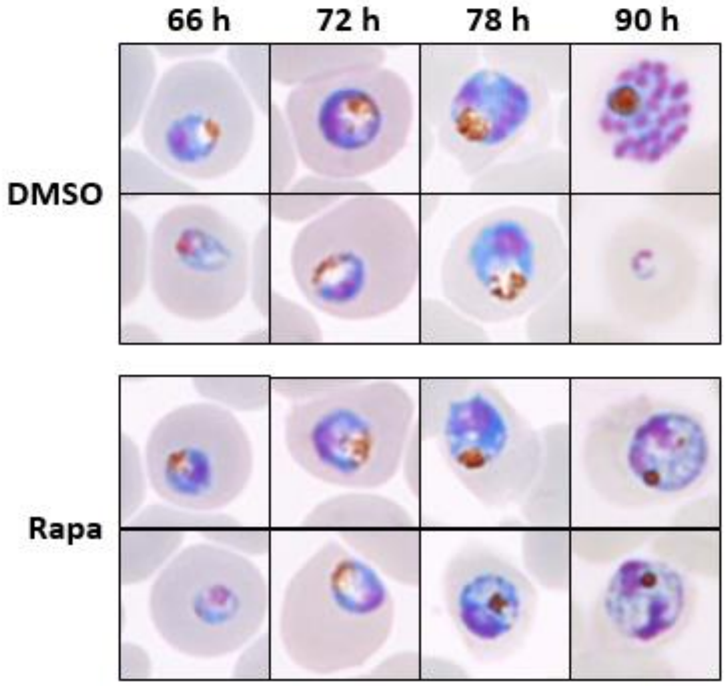

Supplemental Figure S3. Parasite development and morphology when rapamycin was added to synchronous trophozoites at 30 hpi. Parasitemia was determined by FACS and parasite development was followed by coloured thin smears. In rapamycin treated cultures some parasites started to show differences at around 78 hpi (corresponding to 34 h in the second cycle). At the transition to the third cycle, these parasites did not develop into segmented schizonts and very few ring stages were observed.

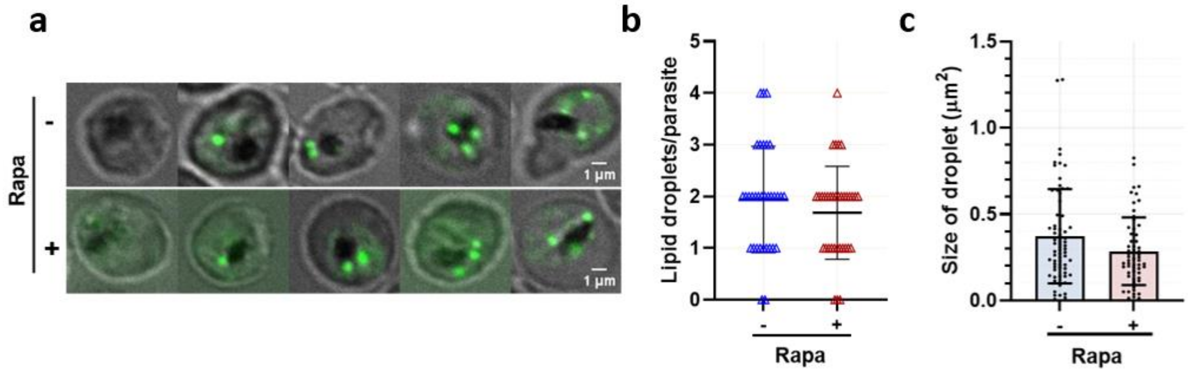

Supplemental Figure S4. iPI3K-ko parasites show no effect on accumulation of neutral lipids. **(a)** Live imaging of iPI3K-ko parasites at 75 hpi after Rapa and DMSO treatment at 30 hpi. Lipid droplets were stained with Nile Red (1 µg/mL). Images were taken on a confocal spinning disc CSU-W1 microscope; excitation 515 nm and emission 585 nm. Shown are merged images of the fluorescent signal and DIC. Scale bar = 1 µm. **(b)** Quantification of the number of lipid droplets per parasite. The graph shows the mean ± SD; n = 35 parasites. **(c)** Analysis of lipid droplet size in both conditions was determined using ImageJ software. Shown is the mean ± SD. There was no statistical difference in panel (b) and (c) using Mann-Whitney test.

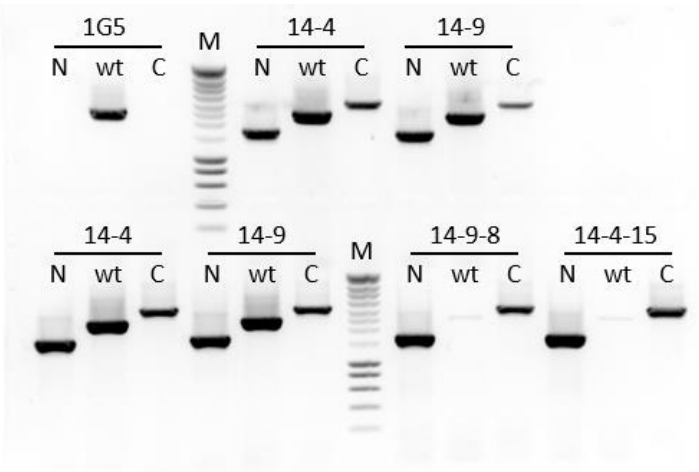

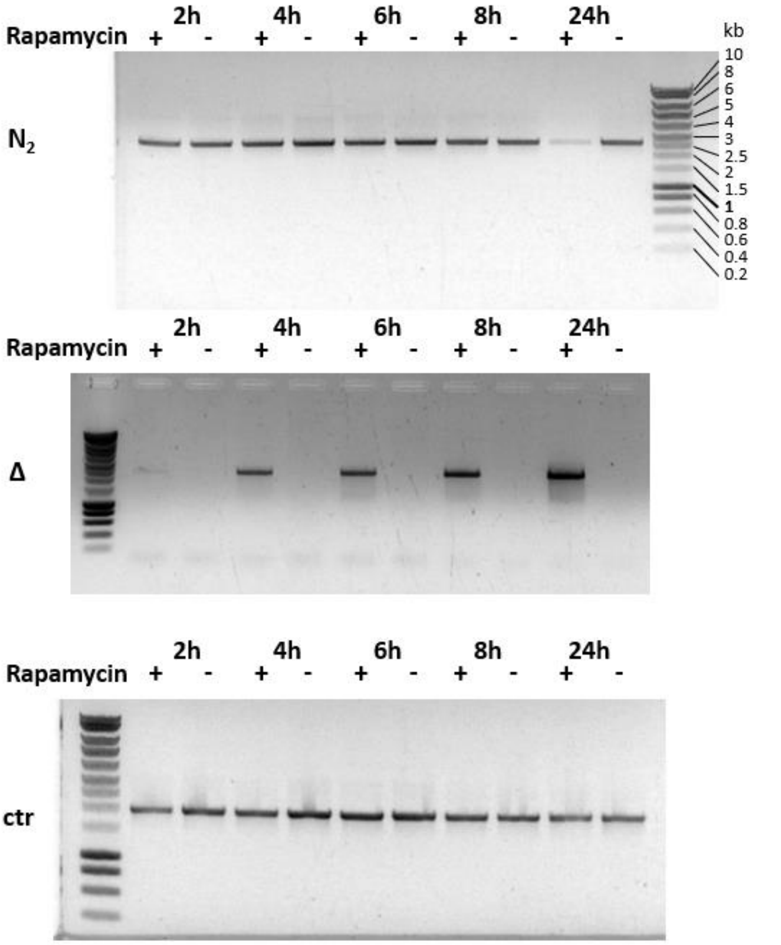

Supplemental Figure S5: Full size images of the PCR analyses shown in Figure 1b (top) and 1d (bottom). DNA size marker SmartLadder (Eurogentec) was used throughout this study and the size in kilobases is indicated.

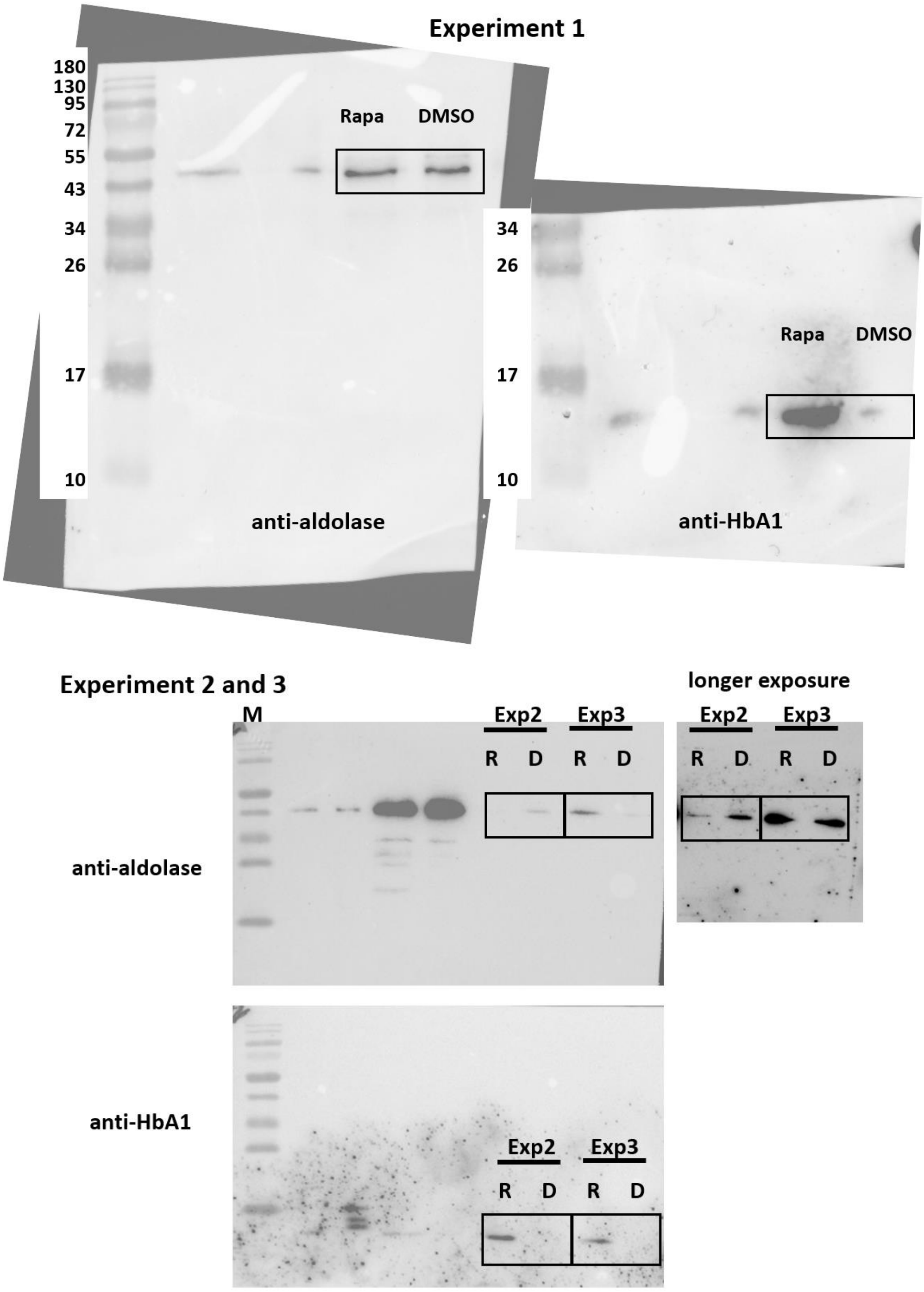

Supplemental Figure S6. Full-size Western blots of Figure 6a. The blots were first developed with anti-aldolase antibodies before development with anti-HbA1 antibodies. The bands shown in Figure 6a are from experiment 1. The blots for experiment 2 and 3 are shown below. For longer exposure times, the membranes were trimmed. Marker bands are labelled in kDa.
